# 10 years on from the landmark stroke thrombectomy trials, where are we now? A qualitative study examining professional views on the implementation of endovascular treatment for ischaemic stroke in England

**DOI:** 10.1136/bmjopen-2025-104126

**Published:** 2025-09-17

**Authors:** Rosemary L Simmonds, Jo Day, Martin James, Phil White, Christopher I Price, Lisa Shaw, Gary Ford, Catherine J Pope

**Affiliations:** 1 Health and Community Sciences, Faculty of Health and Life Sciences, University of Exeter, Exeter, UK; 2 NIHR Applied Research Collaboration South West Peninsula, Health and Community Sciences, University of Exeter, Exeter, UK; 3 Royal Devon University Healthcare NHS Foundation Trust, Exeter, England, UK; 4 Stroke Research Group, Clinical and Translational Research Institute, Newcastle University, Newcastle upon Tyne, UK; 5 Stroke Research Group, Population Health Sciences Institute, Newcastle University, Newcastle upon Tyne, UK; 6 Oxford University Hospitals NHS Foundation Trust and Radcliffe Department of Medicine, University of Oxford, Oxford, UK; 7 Nuffield Department of Primary Care Health Sciences, University of Oxford, Oxford, UK

**Keywords:** QUALITATIVE RESEARCH, STROKE MEDICINE, Health Services

## Abstract

**Abstract:**

**Objective:**

To explore multiprofessional views about system-wide factors influencing (impeding or facilitating) the delivery of stroke mechanical thrombectomy (MT) services and/or improvements to this pathway in England.

**Design:**

A pragmatic exploratory qualitative study using online focus groups and semi-structured interviews with National Health Service (NHS) professionals and those working in a stroke strategic/policy lead role. We thematically analysed the data using the Framework Approach to understand participants’ views on the challenges to improving current and future MT implementation.

**Setting:**

NHS trusts and other key stroke strategic/policy organisations covering 10 geographical regions in England and a national perspective.

**Participants:**

A total of 29 professionals, working in an NHS clinical and managerial position and/or a stroke strategic national/regional clinical/policy lead role, participated in five focus groups and six individual semi-structured interviews between April and June 2024.

**Results:**

We identified five themes relating to MT implementation progress and challenges (1) workforce, (2) clinical care pathways, (3) service/system, (4) cross-cutting theme: communications and (5) cross-cutting theme: culture. Our analysis emphasised the increasing complexity and inter-related factors shaping the emergency stroke pathway for MT provision and a need to acknowledge key people-related, organisational and sociocultural factors during service planning.

**Conclusions:**

Despite the challenges and complexity, professionals were optimistic that further progress would be made with MT delivery in England. However, ongoing improvement strategies are required, which also acknowledge wider cultural factors and system-wide relationships and are not just focused on care pathways and resources.

**STRENGTHS AND LIMITATIONS OF THIS STUDY**

- The use of both focus groups and semistructured interviews with professionals generated rich data (focus groups eliciting broader views and the interviews more detailed perspectives) and we gained feedback on our analysis from a stroke public involvement group.
- The use of abductive (deductive and inductive) coding was a methodological strength, allowing us to build on existing concepts while facilitating the identification of new themes/ideas of importance.
- A limitation for this study was conducting focus groups online, which may have inhibited the spontaneity and flow of discussion but greatly facilitated multiregion participation.
- Participation from and/or further stakeholder discussion with those working at a national policy level could provide additional insights and understanding to the systemic and, in particular, sociopolitical challenges.

## Introduction

 A watershed moment in emergency stroke care arrived in 2015 when randomised controlled trials showed that mechanical thrombectomy (MT) removal of blood clots from large arteries in the brain significantly improved outcomes in acute ischaemic stroke.^1^,^3^ However, implementation has been disruptive for healthcare systems^4^,^8^ as this time-critical procedure, for approximately 15% of stroke patients, requires highly specialised facilities and interventionists that are not available in most hospitals. Furthermore, in England, of 107 acute stroke centres, only 24 provide MT.^9^

Previous studies have discussed the global challenges in establishing and improving thrombectomy services. Schellinger *et al*^10^ identified safety, logistical (including workforce, centres, imaging and geography) and financial challenges, arguing that a high degree of collaboration and well-defined emergency medical service guidelines are crucial for MT implementation. More recently, Opsel *et al*,^11^ described problems such as lack of public awareness of stroke symptoms, need for upfront payments to services, lack of a functioning ambulance system, geographical accessibility and delays in transfers between hospitals, access to/delays in imaging, lack of trained staff and equipment/suites to undertake the procedure and constrained hospital bed capacity. They noted further disparities included the travel distances for some populations to a service, underutilisation in certain population subgroups and the lack of a continuous 24/7 thrombectomy service coverage in a country. A recent review of the effectiveness of strategies to reduce delays in accessing thrombectomy concluded that a direct to comprehensive stroke centre (CSC) approach was the most effective model, but a need to improve selection processes/decision-making in the pre-hospital setting to identify patient suitability for direct transfer to a CSC, travel times and capacity challenges prevent the direct translation of this evidence into practice.^12^

Currently in England, there are 24 hospitals that offer MT treatment, but the majority (74%) of patients are admitted via 83 acute stroke centres (ASCs) where MT eligibility must be recognised through clinical assessment and imaging before rapid secondary transfer to a regional CSC.^13^ Ford *et al*^4^ identified that clinician and organisational communication and cooperation are key for the successful implementation of 24/7 thrombectomy services in England. Other challenges include the availability of the neurointerventionist workforce and agreed standards for triage and patient transfer.^8^ Furthermore, a recent study exploring healthcare professional views about a proposed new pre-hospital thrombectomy pathway in Engand^14^ identified the following complexities involved in implementing and sustaining MT provision: organisational culture, changing patient demographics (age and ethnicity), variable regional healthcare provider systems, time-dependent treatment pressures and a challenging sociopolitical healthcare context.

The MT target in the 2019 National Health Service (NHS) England Long Term Plan was for 10% of stroke patients to receive thrombectomy by the end of 2022.^15^ In the UK (excluding Scotland), 3.9% of stroke patients received MT in the period, April 2023–March 2024,^16^ with a much lower rate among patients who required transfer for treatment.^13^ Although this represents rapid improvement in the percentage of stroke patients receiving thrombectomy since 2019 in England, the current rate is still only just over a quarter of those eligible, so it raises the question as to the progress being made to overcome implementation barriers and how adaptable the system will be for emerging evidence of criteria for treatment and new technology to further enhance outcomes.

To our knowledge, there are no qualitative studies exploring the views of healthcare providers and stroke professionals about progress and barriers to improving patient access to thrombectomy and future system readiness. As qualitative research methods can elicit rich, contextual information, we anticipated building on previous studies to further illuminate understanding on improving MT treatment provision given the limited progress with implementation in England. Therefore, this study sought to explore multihealth professional views about system-wide factors that can influence (impede or facilitate) scaling up the delivery of emergency stroke MT treatment for acute ischaemic stroke to inform future implementation and improvement work.

## Methods

### Design

We conducted a pragmatic exploratory qualitative study using on-line focus groups and individual semistructured interviews to capture views from a broad range of multihealth and stroke professionals. This included NHS clinicians and non-clinicians in operational, managerial and strategic emergency stroke care/delivery and policy roles. The qualitative researchers (JD: social psychologist, RLS: social scientist, CJP: medical sociologist) were non-clinicians with a range of complementary interests, skills and experience in applied health services research along with co-investigators with expertise in stroke service delivery and research. We drew on our knowledge from the implementation and improvement science fields which promote the adoption, integration and optimisation of evidence-based innovations in healthcare services.^17^ The study is reported according to the Consolidated Criteria for Reporting Qualitative Research.^18^ Approval to conduct the study was given by the Health Research Authority (21/HRA/4859) and University Ethics Committee (492029).

### Sampling and recruitment

Purposive and maximum variation^19^ was used to inform the sampling and recruitment of professionals working in NHS stroke clinical and managerial lead positions across ambulance service, emergency care, acute and CSCs in hospital trusts and wider regional and national strategic/policy organisations across regions in England. In discussion with co-investigators (CP, MJ, PW, GF, LS), who have expertise in emergency stroke care and knowledge of formal and informal networks relevant to thrombectomy provision, we identified a range of roles and regions. This indicated that at least 20 participants would be reasonable within the study resources. We monitored sampling during data collection and ceased recruitment once we achieved rich and meaningful coverage.^20^

Potential participants were identified by the co-investigators (CP, MJ, PW, GF, LS) and they distributed an email invitation prepared by the qualitative researchers. Interested potential participants were asked to respond to the email, either directly to the qualitative researchers, whose details were provided within the invitation or to the initial contact who then forwarded the positive response to the qualitative researchers. The qualitative researchers subsequently provided further information about the study including a participant information sheet and potential dates/times for the first planned focus group. Individuals were asked to reply to confirm willingness to participate and agree meeting arrangements. The option for an individual interview was discussed if dates/times for the focus group were inconvenient. If there was no reply after 2 weeks, follow-up of contacts ceased.

To minimise time burdens, the primary method of consent was verbal, although written consent forms were used, if preferred by the participant. At the start of each focus group/interview, a researcher read out a consent script consisting of eight statements and each participant was asked to state verbally that they agreed to each statement or not and then provide their overall consent to taking part in the study. The MS Teams remote software recording function was activated immediately before the verbal confirmations to record this consent process. Participants were offered an optional £30 voucher for taking part.

### Data collection

We undertook the online focus groups and individual interviews using a semi-structured topic guide to inform discussion (see online supplemental file for example questions). The questions were designed by all the co-investigators to explore professionals’ views on: (1) the largest system-wide challenges to expanding MT treatment and what is most needed to optimise patient access, (2) general workforce issues, (3) clinical care processes. for example, rapid access to brain imaging and early review by a stroke specialist, (4) service models and improvements for access to MT and how they might work, (5) financial viability, investment priorities and other service developments impacting the availability of resources, (6) what is needed for a sustainable model of MT provision across the NHS and (7) any other challenges and possibilities.

### Data analysis

The digital recordings of the focus groups/interviews were transcribed verbatim and deidentified. NVivo Qualitative Software package was used for the management and analysis of data. The transcripts were analysed abductively using the Framework approach^21 22^ by RLS, JD and CJP and discussed with all co-investigators as analysis progressed. First, familiarisation of each focus group and interview transcript by summarising key points. Next, a coding framework was developed informed by health delivery performance domains^23^ and the identification of additional codes. All transcripts were then coded in three stages: (1) deductive thematic coding to the following health delivery performance domains: workforce, financial viability, clinical care, service model; (2) inductive thematic coding of cross-cutting ideas/issues identified throughout the deductively generated data. All the coded data themes were further analysed and summarised by charting in MS Word tables; (3) the ‘thematic networks’ approach^24^ was used to further analyse and identify themes cross-cutting the deductive data; this is a qualitative research tool for identifying and interpreting patterns of meaning and levels of abstraction within the data by creating visual representations (network maps) of the relationships between themes. We produced network maps for two identified cross-cutting themes: communications and culture.

Rigour in analysis was achieved through double coding of purposively selected transcripts and from researcher immersion in the data and charts. Triangulation of the findings was undertaken through discussions between the qualitative researchers, with all co-investigators and then through a presentation and discussion of initial findings with members of a stroke survivor group. Minor discrepancies were discussed, and no significant disagreements needed to be resolved.

### Patient and public involvement

A stroke survivor contributed to the initial study design and, as noted above, we shared and discussed initial findings with a stroke survivor group that shaped the interpretations presented in this paper.

## Results

The focus groups (n=5) and interviews (n=6) were conducted between April and June 2024 with 29 professionals from 10 regions in England (table 1). The duration of focus groups ranged between 58 min and 1 hour 45 min and for interviews, it ranged between 45 min and 56 min. The data are presented below by the following five themes—three deductive (1) workforce, (2) clinical care pathways, (3) service/system and two inductive cross-cutting, (4) communications and (5) culture.

**Table 1:** Participant characteristics

Professional role	Participated n=29	Region in England
Clinical and Managerial leads in Ambulance services and hospitals who are involved in or take an interest in delivering emergency stroke care	5	Northeast, Northwest, Southeast, East Midlands
Professionals with knowledge of commissioning of acute stroke care	3	North of England, Midlands, East of England
Stroke consultants and nurses at CSCs and at ASCs	9*	East of England, Northwest, Northeast, London, Southwest
Interventional neuroradiologists	3	Northeast, Midlands, London
National and regional strategic stroke care/policy role, for example, integrated stroke delivery networks	10*	East, West and North of England, National

* One participant in two roles

ASC, acute stroke centre; CSC, comprehensive stroke centre.

### Workforce

I think the workforce issue is the biggest burning platform around optimal clinical effectiveness and delivery of interventions. (ID09)

Current and anticipated workforce issues were considered critical by participants for further increasing the rate of MT. Staff shortages and ongoing education/training posed both challenges and opportunities for all service providers along the whole acute stroke/MT pathway, and the ubiquitous nature of this issue was further highlighted:

I think there’s been an error in assuming that this is about the number of INRs… obviously, that’s massively important, but (also)…the ‘front-door assessment’, via nurses, junior doctors, radiologists, radiographers—you name it—the whole pathway. (ID09)

Working patterns, comprising ‘out of hours’ shifts and inequitable remuneration for being on ‘standby’ were viewed by participants as negatively impacting work/life balance. These factors were also thought to affect the onward recruitment and retention of interventional neuroradiologists (INRs) who deliver MT treatment. This is compounded by the upscaling of MT to 24/7 provision.

Linked to addressing the current/projected shortage of INRs and workforce training more generally, participants discussed the merits of the General Medical Council credentialing process to provide fast track training to doctors from non-radiology disciplines. In support, participants with a national strategic remit pointed to the fragility of the current ‘super-specialisation’ of the role contributing to shortages of INRs and difficulty in filling job vacancies. The positive effects of the training and supply of INR numbers were also reported:

We’ve got 38 registrars in training at the moment for INRs. So, in terms of future workforce for the INRs, I feel that the future, in that sense, is bright. So, I think there should be enough INRs as time goes on. (ID23)

For pre-hospital services, participants discussed staff shortages due to a high staff turnover, which can impact on the functioning of the service to ensure rapid ambulance transfers. Stroke as a topic was thought to be underplayed in ambulance personnel training and dedicated, paid time for mandatory stroke education and professional development was recommended. Although this was an issue for general acute stroke care, it was thought to have direct relevance for MT, which sits on a platform of an emergency response for stroke of which pre-hospital services are an integral part.

### Clinical care pathways

Access to diagnostic tests, particularly in the pre-hospital setting, and scans was raised as a significant issue currently affecting the efficacy and efficiency of the MT pathway. At present, there are no definitive tests for assessing types/severity of strokes in the pre-hospital stage:

What would make the difference…is reliable pre-hospital prediction of who’s likely to have a large vessel occlusion and, therefore, would benefit from thrombectomy. Because, with that, we could then develop a model that’s similar to what cardiologists do for their PCI…you could selectively transfer people to the Centre where they’re going to get the treatment. (ID06)

Participants reported possible diagnostic approaches that could be used pre-hospital such as a possible composite score based on clinical parameters and biomarkers. The use of mobile stroke units and their efficacy was also discussed with reports, from countries outside the UK, of positive impacts on MT numbers, door to needle time and reduced levels of disability for patients post procedure. The need for reliable pre-hospital diagnostics to distinguish different types of stroke and reduce the numbers of non-stroke ‘mimics’ was proposed by participants as potentially ‘game changing’ for the efficiency/efficacy of the MT pathway.

Within hospitals, the issue of how to accelerate and enhance the effectiveness of clinical, diagnostic imaging and interpretation in ASCs was raised in relation to artificial intelligence (AI) software, machine learning and improved co-ordination between ambulance and hospital services. Imaging and diagnostics were discussed as enhancing the speed of the MT pathway via an ASC referral as follows:

So the magic wand would be, could we get the patients to have the right imaging in the acute stroke centres, (so) we can encourage more referrals into the comprehensive stroke centres… (ID28)

A stroke clinician discussed innovative approaches to the interpretation of scans, such as AI and machine learning, and their experience of the merits and drawbacks of these approaches. They argued that currently, AI systems and software are ‘sub-optimal’ and necessitate a human ‘trained eye’ to interpret data on CT or MRI scans.

### Service/system

Common issues were identified regarding the service/system MT pathway available across England. How these issues play out in each region differs due to patient flow, financial viability, particular geographies (urban or rural), the location/accessibility of CSCs and how well the services are networked/coordinated. Participants generally viewed that the number of stroke centres in England is about right and the focus should be on optimising the existing models.

#### Patient flow

Participants explained that a fast-track approach of rapid diagnostics at ASCs while an ambulance is waiting and onward transfer to a CSC if needed could reduce delays before MT but would depend on whether ambulance service trusts could protect the vehicle for this possibility due to wider service pressures. An ambulance service lead reflected on the realities of how they organise crews and onward transfers. For some ambulance trusts, the operations centre management systems for deployment of crews are based on fairly strict time/task based criteria and tracking of vehicles so they can be made available for further transfers:

Well, for us as a trust, our preference would be that we dropped the patient at A&E, so to speak, they could go and have the scan or whatever and then when it’s identified that they want an onwards transfer, they book that as a new transfer. (ID26)

As illustrated above, the operational needs and targets of the ambulance service are not necessarily compatible with those of an optimal MT pathway where the ‘retention’ of crew, for possible transfer to a CSC, is considered an important time saving clinical goal and has been successfully implemented in other ambulance systems such as Dublin, Ireland. However, the current pressures facing ambulance services were perceived by participants to impact on the availability of transfers generally:

I think when there are problems, you go downstairs to A&E sometimes and you see that there’s a huge backlog of ambulances trying to get into A&E, and you say: “where’s the ambulances to try and transfer these patients?” A lot of them is because they’re outside the hospital, trying to get in. (ID23)

When hospital Accident & Emergency (A&E) departments experience extreme pressure of demand with ambulances queuing outside hospital A&E departments for general admissions, the inflow and outflow efficiency of the MT pathway is also impacted. Post-MT procedure, rapid repatriation by ambulance to a local stroke centre is required to enable more stroke patients to be admitted and treated, but ambulances are less available under situations of heightened demand.

The challenges of increased demand on ambulance services and hospital A&E departments, a lack of hospital bed capacity and staff shortages were seen to negatively impact on the capacity of the overall system to provide 24/7 stroke/MT treatment.

#### Geographies and demographics

Geographical variations in the configuration of stroke service models/MT pathways across England were discussed at length as impacting on equity of patient access to MT treatment. Regional demographic variations were also viewed as contributing to ‘huge’ variation in access to MT. A sharp contrast between the north and south of England was noted with people living in London having the best direct access to thrombectomy. The significance of this was reflected on by one participant as:

…really poignant and profound…and something we really want to improve. (ID27)

Although the number of stroke centres was viewed as generally about right, one participant questioned the provision in coastal/rural areas alongside an increasing older demographic:

Have we got the location of our CSCs right and do we need to consider whether we need more provision in coastal and rural areas to deal with the changing demographics and the changing older population with greater frailty and more risk of stroke (ID29)

Discussions on the topic of financial viability of the MT pathway identified that perceived geographical inequalities in capital funding for MT delivery were a challenge for some regions in England where there is insufficient money to support the structural work required to create a new thrombectomy suite. Issues were also highlighted in relation to private finance initiative (PFI) hospitals:

Lack of capital funding in particular, was holding back the roll-out of thrombectomy…but what’s becoming clearer…is that actually the capital funding for the kit, for the biplanes, is already made available, but one of the barriers is that in PFI hospitals…any building costs are hugely expensive…just fitting out the suite is going to cost anywhere between three to six million pounds. (ID27)

#### Hospital stroke/thrombectomy networks

Participants reported key issues that hospitals face in setting up and providing MT treatment. Participants discussed how to support particularly INRs when setting up non-neuroscience MT centres to ensure that teams are not isolated from their peers and are able to keep abreast of developments in the field. To improve referrals and support of MT services, participants recommended making more use of regional clinical networks, such as integrated stroke delivery networks, to share expertise and find solutions to common issues.

Suggestions for improving referrals for MT from ASCs were made, such as thrombectomy hospitals sharing facilities and staff resources over a 24/7 time period. This approach was thought to be best suited to non-rural geographical locations where thrombectomy hospitals are within reasonable transfer times of each other. Some participants also reported that adopting an Irish service approach could improve repatriation of MT patients from CSCs to ASCs in rural/remote locations:

What would optimise things…is the Irish model, which is a ‘door-in, door-out’ policy. So, what happens in Dublin is that…a doctor goes in the back of the ambulance with the patient. They stay. They have the procedure. If they’re stable, they go straight back (ID13)

Participants thought the ‘treat and return’ model could also be used to address bottlenecks at thrombectomy hospitals, which at weekends can be inundated with non-stroke admissions and clinics. Same day repatriation to referral centres could also improve the inflow/outflow ratio and free up hospital beds to support thrombectomy treatment.

Despite the challenges highlighted above, participants in national strategic roles argued that positive progress was being made to meet MT targets, for example, imaging occurring more quickly, ambulances staying with patients and more centres delivering 24/7 MT services.

### Cross-cutting theme: communications

**Figure 1:**
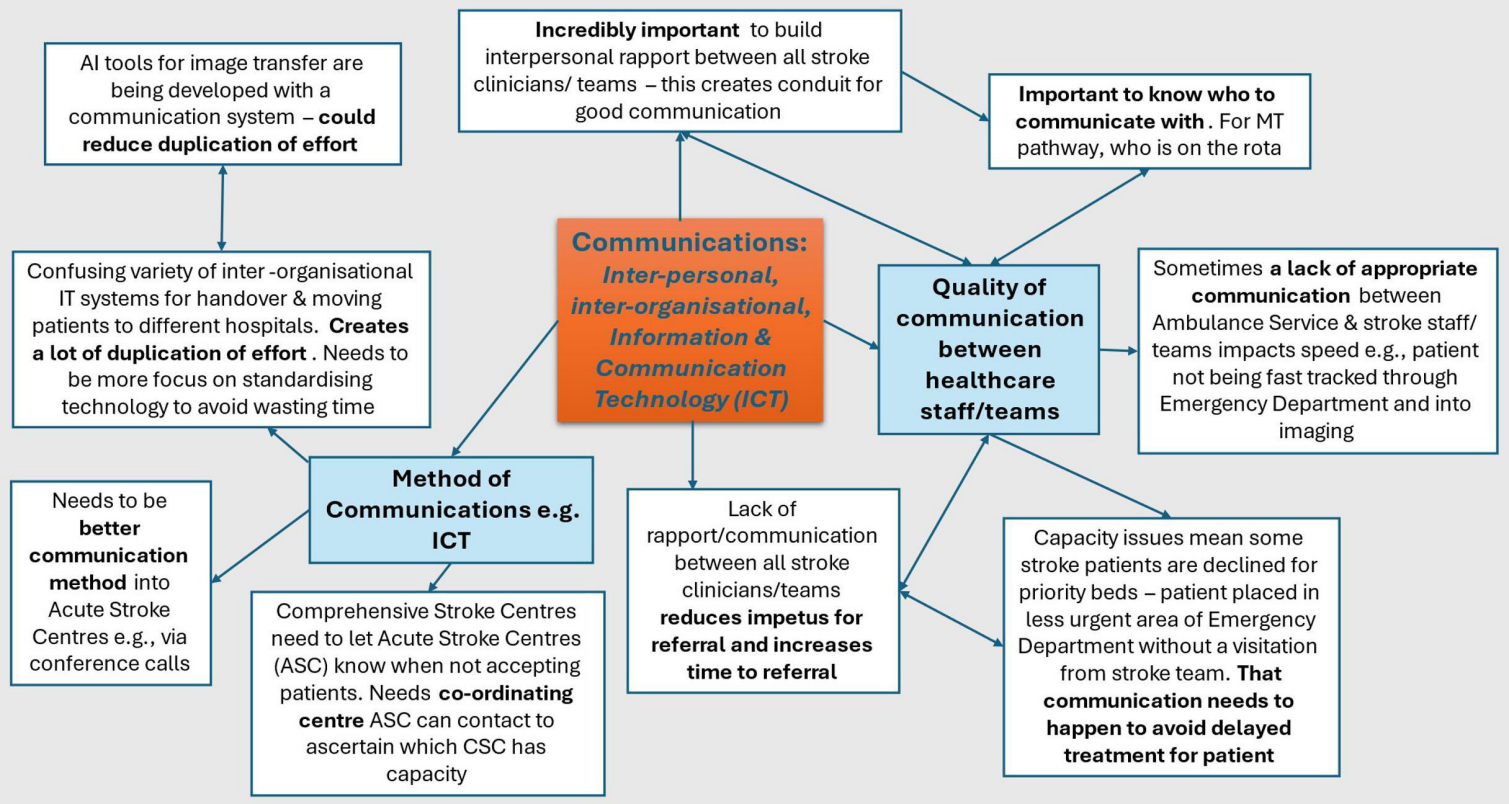
Network map for cross-cutting communications theme. CSC, comprehensive stroke centre; MT, mechanical thrombectomy; IT, information technology.

The communications theme (figure 1) is an inductive theme that cross-cuts the previous three deductive themes and captures how the quality and method of communications affects the efficacy of the MT pathway.

An ambulance service clinician shared their experience of delayed communication during handovers of patients to hospital stroke teams and highlighted the importance of pre-hospital decisions about the best destination for the admission. They explained how the urgent response breaks down if there is ineffective or indirect pre-hospital communication with the stroke team, especially when there is a lack of room on stroke units for rapid assessment of new admissions. They reported that this situation is improved in hospitals where stroke teams meet the paramedics for a handover in A&E to reduce delays as much as possible. This point was echoed by the strategic lead:

If there hasn’t been appropriate communication between the paramedics and the acute stroke team in advance, then they’re not fast-tracked through ED [Emergency Department] into imaging. (ID29).

Ambulance service participants also emphasised the need for polite, professional and efficient verbal communications between ambulance crews and hospital stroke staff. Although they acknowledged the pressures being experienced by hospital stroke teams, they pointed out that both sets of professionals are in a ‘relationship’ which is about working together to ensure the stroke pathway is working in the best interests of the patient:

You might have these sorts of systems that are quite sleek and they should work…we've come up with this amazing model and but when you then put it into practise like ‘oh, why isn't it working?’ Oh, because the crews don't like talking to the stroke nurse because once they were mean to them or the signage didn't show them where to go. (ID24)

The value of using a structured format, such as the Situation, Background, Assessment, Recommendation (SBAR) to improve the provision of information during pre-alerts/handovers by emergency services to hospital clinicians was suggested.

Challenges with the method of communication were raised by ambulance service participants who shared that poor connectivity and the use of telemedicine in rural areas of England could be difficult. They advised against a heavy reliance on innovations/systems based on access to digital connections, as they were perceived as being insufficiently robust.

…so some rural areas…just because of where they are, they don’t always get the best signal. (ID26)

Hospital stroke professionals related their experience of frustration at the time taken to contact different thrombectomy centres with capacity to accept a patient. Participants proposed possible solutions to this problem based on a more centralised/coordinated approach including the potential for conference calls:

That’s why some form of centralised system, to let you know immediately on your phone, which Centres are open and which not. They’ve got it in Berlin. (ID14)

It’s really basic stuff…our local Centre can’t give us a direct-dial number, because the ‘Consultant of the day’ has a different direct (number) each day. So, we end up going through ‘switch’ (switchboard) which is just bonkers really! I mean you should be able to pick up the phone, have a conference call with the local receiving Centre, the Stroke Consultants and the INR patched in, pretty much immediately. (ID22)

Several issues were raised around problems encountered when working within healthcare systems equipped with incompatible IT systems. Some participants identified AI solutions for potentially reducing duplication and the effort of information input and retrieval. The quote below emphasises the importance of communication for building rapport and aligning teams to speed up the MT referral process.

Communication is incredibly important between all those involved…part of that is actually knowing who to communicate with…having rotas and knowing who’s on the rotas and things like that. So, once you’ve actually built up that rapport, you know, between all the teams, stroke physicians, stroke coordinators, nursing staff, IRT coordinators, consultants…who to contact, then actually, it reduces the time to referral very quickly. (ID22)

### Cross-cutting theme: culture

**Figure 2:**
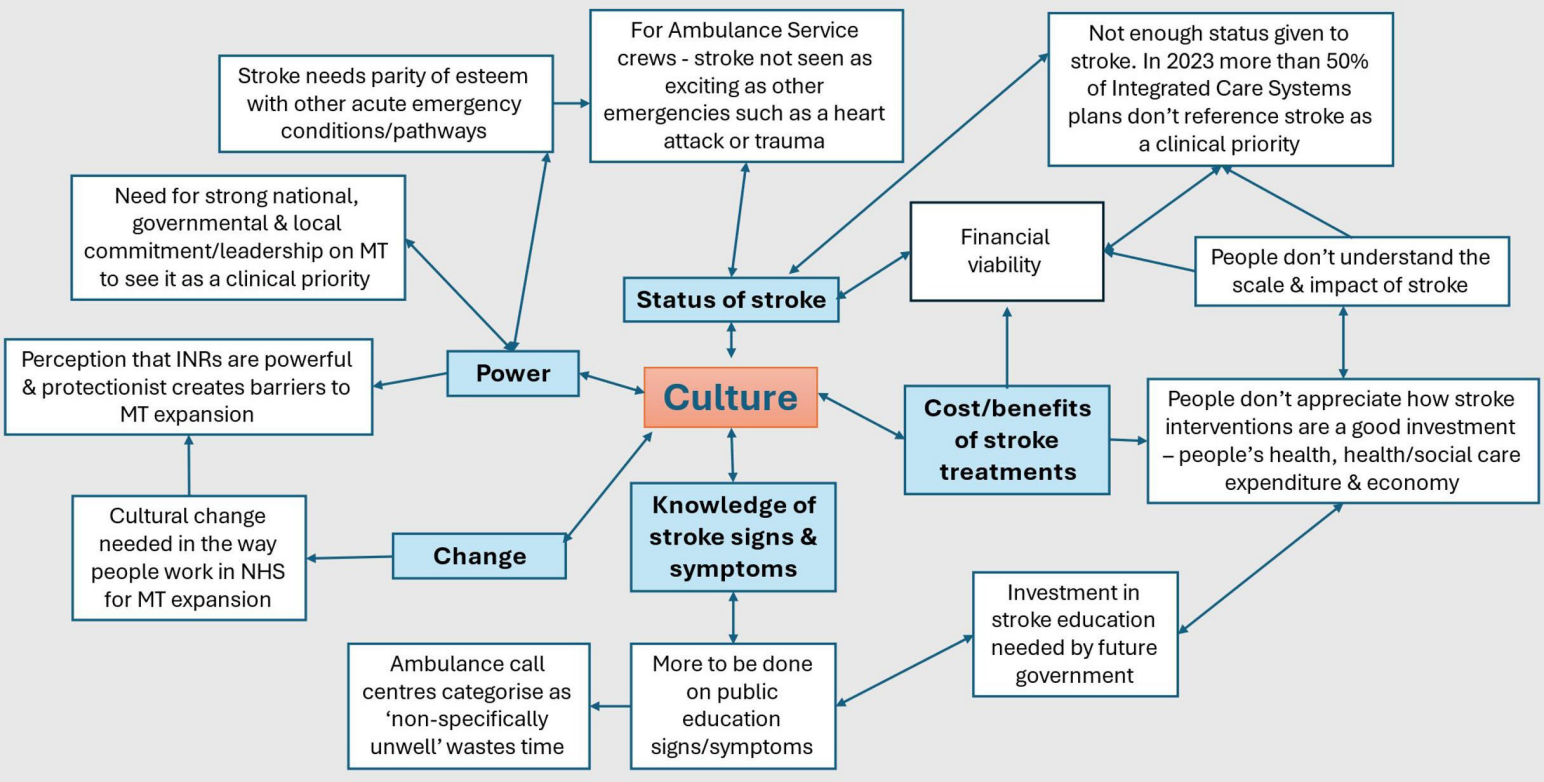
Network map for cross-cutting culture theme. INR, interventional neuroradiologists; MT, mechanical thrombectomy; NHS, National Health Service.

The second inductive cross-cutting theme of culture (figure 2) captures how stroke and stroke treatment is perceived and the status it is given in health services and societally. Participants reported that stroke is neither viewed as a priority nor a condition that matters—either by the public, the media or politically. Public education and awareness-raising of stroke and acute stroke treatments was considered an ongoing need. Within health services, participants argued that stroke is viewed as less important than other acute conditions which can inhibit positive changes, such as including new diagnostic technology in ambulances:

When we were talking about the microwave technology and such like, and it fits into…‘making stroke sexy’, you know. A place where people want to work and such like. If the ambulance [service/personnel] don’t see stroke as being important…which they don’t because the only three things in the ambulance service that’s important is cardiac arrest, myocardial infarction and major trauma. After that, everything else is very much, you know, in a second echelon of response. It doesn’t matter what future pieces of kit are out there for people to use, the ambulance service won’t put them onto a vehicle. (ID17)

Cultural perceptions of stroke across hospital trusts were discussed and how this is thought to contribute to inequalities of access to clinical care, such as diagnostic scanning and lack of parity of esteem with other injuries/conditions:

It’s really clear across many hospitals in the country that there’s not parity of esteem across stroke pathways compared with say trauma or cardiac pathways and …a ‘brain attack’ being as important as someone who’s fallen, you know, three foot off a ladder and the stroke patient gets bumped off a CT scanner, while someone else has a whole-body CT for an innocuous injury. (ID09)

Furthermore, a strategic lead reported how stroke is not referenced as a clinical priority in half of integrated care systems (ICS) plans. The extract below illustrates perceptions of the status of stroke at all levels in the system from national to local:

I think we need a cultural transformation that identifies that people with thrombectomy have a high chance of a response, under certain circumstances and anatomies. They should be treated as a brain attack, cardiac arrest, major trauma priority. (ID04)

If stroke is not perceived as a priority at a cultural level and imbued with less ‘power’ than other acute conditions, it does not follow that power struggles are absent within this specialism. In relation to the INRs as a profession, some participants shared the perception that ‘professional protectionism’ is at play in some regions/hospitals in terms of expanding the MT service:

I think what saddens me…there are really keen cardiologists around… there is one in our region who’s done all the courses. He’s got every kind of qualification, but they can’t get a Centre to adopt them…I think that’s just kind of criminal, when you’ve got someone that keen, who’s got the skill…you’ve got that professional protectionism that won’t invest in that person and ensure that they can be part of the solution. (ID18)

The quotation above illustrates some resistance to change within the INR profession, through expanding the numbers of INRs by offering non-traditional routes to qualification. Furthermore, the suggestion of professional protectionism at play among the INR profession could be applied to the following extract where it is argued that the only people who can perform a MT safely on patients are INRs who have followed the traditional route to qualification:

I think if the INRs are telling us they find it [MT] really hard and they think the only people that could do it are the people who are trained in IR and then neuro-IR…they think I’m the only one who can do it the best…when you’re there with the catheter in someone’s brain…I think those are the people who should be telling us who should be doing it. (ID19)

Others viewed this as about ensuring that professionals are trained and undertaking thrombectomies at a volume to continue to deliver safely to patients. Professional cultures and power imbalances across clinical specialisms can be perceived to pose a significant barrier to making changes needed to improve access/time to thrombectomy treatment:

It’s always people, they are our biggest asset and also some of the inhibitors of change and I’m not sure we’ve thought about that in a bigger way. (ID28)

Participants argued that knowledge of the positive cost benefits of MT is needed from the ‘bottom up’. To better support the financial viability of MT, decision-makers and budget holders in the system should be aware of stroke MT treatment and cognisant of the positive cost benefits:

Well, a lot of people haven’t heard of thrombectomy, and this includes system decision-makers—ICB [Integrated Care Board] leaders, for example. (ID29)

Inherent historical and regional organisational variations in the current funding models for MT were discussed by participants. The complexity of this is illustrated in the following quote:

There’s a lot of ‘smoke and mirrors’ in the system at the moment around funding for thrombectomy…we’re not like a lot of the surgical specialities that have tariffs and business units and if you made a profit, you’d plough it back…because most stroke services historically have been part of geriatric medicine…So, it all gets consumed relatively quickly in the abyss…the only bit of the stroke pathway that’s inspected is thrombectomy, nothing else. So, it’s totally separate at ICS (Integrated Care System) level and regional and national level, around its funding…there’s been a little bit of work done looking at the thrombectomy tariff but…that tariff goes to Neurosciences Centres…members of staff in an ASC, make the diagnosis, do the imaging, arrange the transportation and get the patient to the CSC and then the tariff goes directly to the CSC…the funding pathway doesn’t serve anyone as it currently stands. (ID09)

The importance of leadership emanating from the top at the political level and throughout the different system levels of the NHS to overcome some of the challenges discussed in this theme was emphasised by participants with a strategic role:

…it just goes back again to leadership, you’ve got to have everybody lining up to deliver on a big, hairy, audacious goal that is thrombectomy. But if everybody lines up and you’ve got leadership at all the different levels of the system, you’ve got people collaborating, you’ve got great trust and relationships, it can happen. (ID29)

## Discussion

We explored regional and national multiprofessional views on the system-wide factors influencing the implementation of MT in England. In our analysis, we identified two important cross-cutting themes of communications and culture. These were perceived as important influential factors for improving rates of MT treatment at local, regional and national levels in England. Additionally, influential factors relating to the workforce, clinical care/patient pathways and services/systems were identified. Our study highlights the complexity of improving MT access in England due to the interdependencies of these factors.

We identified important links between cultural and sociopolitical influences, the status of stroke and the associated power and leverage to make changes happen. For example, the clinical/patient and socioeconomic benefits of effective treatments such as MT continue to require a higher public, professional and organisational profile.^25 26^ Giving higher priority to stroke should also help achieve equivalent status between stroke and other time-critical conditions such as MI and trauma. In relation to expanding the implementation of MT, some of the barriers reported were typical of implementation of complex interventions, including organisational culture, support from leadership/management and factors such as trust and collaboration—are identified as important elements in how systems function.^27^ Participants argued for strong leadership, at all system levels, to assertively promote stroke as a priority condition which would lead to a return on the investment of time and resources. System leadership, additional investment and a coordinated response have been identified as crucial for improving stroke services in England.^28^ Our analysis confirms the importance of recognising these wider contextual factors in strategic planning as they were strongly implicated as creating barriers to expansion of MT treatment.

Within the care pathway, the importance of communications was raised by participants. Structured formats, such as SBAR, were considered potentially useful particularly for handovers/transfers between services. There are multiple tools that could be used to structure communication to improve clinical care pathways; a stroke-specific one was valued by ambulance service clinicians in a study to increase thrombolysis rates.^29^ Two systematic reviews on the efficacy of the SBAR tool observed a lack of high-quality research on this widely used communication tool,^30^ and future efforts to improve communication using SBAR should first confirm high fidelity uptake in clinical settings rather than assuming this has occurred.^31^

Improved methods of communication and AI were considered enablers for better communication between professionals. The importance of clear, polite, professional and respectful interactions and longer-term rapport building to underpin the acute stroke treatment pathway was emphasised. This aligns with previous research identifying that effective communication between and within teams promotes wider cooperation and coordination^25 32^ and could be improved by promoting a teamwork approach.^33^ For example, ambulance service participants viewed giving feedback to paramedics, about the health status of stroke patients, as helpful for promoting a team approach, improving their knowledge of stroke and potentially reducing time to MT.^12^ Referring to one region in England that redesigned acute stroke pathways to implement a 24/7 MT service, Ford *et al*^34^^[p3]^ concluded ‘Communication was key throughout, and the service could not have worked without the cooperation of many specialties.’ This analysis indicates that clinical communication is an essential factor to consider in MT service improvement planning.

Workforce shortages within MT stroke care reflect the wider challenge of a high level of vacancies in healthcare in England.^35^ The insights from our study highlight particular issues recruiting to stroke and MT-related jobs and a perceived poor work/life balance compared with other medical specialities. Although there is optimism of a future increase to the numbers of the neurointerventionists, extending the MT service is likely to require an increase in all staff involved in the acute stroke treatment/MT pathway.^36^ We therefore recommend that addressing the workforce shortages and improving working patterns and providing fair and equitable remuneration is critical and a priority for improving the implementation of MT.

The funding mechanisms for MT were viewed as needing adjustment so they are delivered directly, fairly and proportionately to CSCs and ASCs alike. Capital costs pose a challenge, for example, funding needed to set up thrombectomy suites, as they are not included in the remuneration model for MT. The message from participants was clear on how regional stroke pathways should be configured around each MT centre, that is with sensitivity to regional geographical and demographic contexts as no one approach will fit all. Previous research has indicated that planning over a larger geographical area could be more beneficial for patient access to MT.^37^ Some participants advocated a ‘fast-track transfer’ ASC to CSC model with ambulance services waiting with patients at ASC for diagnostic imaging and a transfer decision to be completed. This should be accompanied by national rapid repatriation policy/agreements, although additional resources may be required to maintain this rapid cycle of ambulance activity.^38 39^ Some participants highlighted direct admission models for selected ambulance patients in well-defined localities, such as that introduced into Stockholm.^40^ In England, the Specialist Prehospital Redirection for Thrombectomy (SPEEDY) cluster randomised controlled trial is currently evaluating a two-stage selection process for direct CSC admission and aims to describe the implications for the whole population with suspected stroke as well as the effect on thrombectomy delivery.^41^

### Strengths and limitations

This study is unique in providing rich, detailed views of system-wide factors across regions and services from multiple professional perspectives. The methodological strengths were the use of both focus groups (elicited views on a broad range of topics), individual, semistructured interviews (allowed for more focused and detailed views from particular perspectives) and abductive analysis to build on pre-existing concepts/ideas. A limitation was with conducting focus groups online, which may have inhibited the spontaneity and flow of discussion. It would have been beneficial to have gained further nuanced views and stakeholder discussions of the findings with a wider range of professional stakeholders across England, including policy representatives.

### Future research and implications

As the MT pathway can necessitate a more complex and interdependent emergency stroke care pathway,^38^ we propose future research adopting a social systems theory approach^42^,^44^ to further understand these complexities and interdependencies. A social systems approach could help identify the influence of socio-cultural factors in shaping systems of care while also complementing findings from mathematical simulation (modelling) approaches to increasing MT provision.^45^ The practical implications from this study suggest that more focus is needed on the influences of communications and culture and, if addressed, could stimulate further implementation progress. We propose that alongside addressing the logistical issues around finance, workforce, ambulance protocols and pre-hospital diagnostics, there is an additional need for innovative improvement approaches focusing on sociocultural, organisational and people factors, to further improve/increase patient access to this evidence-based disruptive innovation.

## Conclusion

We provided a qualitative exploration of multihealth professional views on the system-wide influences on the delivery of MT services in England. We identified that professionals considered that progress has been made with optimism about the future. Our study adds to previous knowledge, identifying the complex, inter-related health system-level issues and critical interpersonal, organisational and sociocultural factors influencing the acceleration of the delivery of MT. The importance of communications and cultural influences highlights a need for disruptive improvement approaches, in some places, to increase patient access to thrombectomy treatment.

## Supplementary material

10.1136/bmjopen-2025-104126 online supplemental file 1

## Data Availability

Data are available upon reasonable request.
