# Supplementary material for: 10 years on from the landmark stroke thrombectomy trials, where are we now? A qualitative study examining professional views on the implementation of endovascular treatment for ischaemic stroke in England
Source: BMJ Open. 2025 Sep 17;15(9):e104126. doi: 10.1136/bmjopen-2025-104126 (PMC12458695; doi:10.1136/bmjopen-2025-104126)
Supplement: online supplemental file 1 [file bmjopen-15-9-s001.docx]

Focus Group and Interview Topic Guide Example Questions

General:

1. What do you consider to be the largest system wide challenges in the current model for providing thrombectomy at the goal of 10% of stroke patients (as per the NHS Long Term Plan)? *If you had a magic wand, what is needed to optimise pathways to patients getting thrombectomy? What do you think are the largest system issues in the current care model?*

Workforce domain:

2. What are the general workforce issues for the current model for providing thrombectomy? *Probe the different issues for hospital and ambulance professionals*

3. What would be the workforce implications if a prehospital re-direction pathway for thrombectomy was implemented? *Probe impact on volumes of work and sustainable hospital stroke services; views on the upkeep of emergency clinician stroke skills in local stroke units if emergency admissions were reduced, how impact ambulance services*

Clinical care domain:

4. What are your views of the current particular challenges relating to the clinical care process for thrombectomy treatment? E.g. Rapid access to CT angiography (CTA), early review by a stroke specialist to confirm patient is possible candidate for thrombectomy

5. What changes in the clinical care processes would be needed if a pre-hospital redirection pathway was introduced? *Probe for different services*

6. If suspected stroke patients are redirected to thrombectomy centres instead of their nearest hospital, some will have non-stroke conditions, what do you think about non-stroke, ‘false positive’, patients being re-directed to a CSC?

Service domain:

7. What are your views about centres providing a 24-hour service? What role is there for more day time/part time thrombectomy centres? (e.g. Monday to Friday 8am to 4pm)? *Probe views for/against, how make this service provision work*

8. Should there be a distance or a time limit on how far patients travel to access thrombectomy? *Exploring the time/geography that seems reasonable e.g. upper limits*

*Probe other service improvements e.g. networking; facilitate 24/7 by recruitment to fill key rota gaps; implement 24/7 regardless of workforce gaps*

9. If a pre-hospital redirection pathway was introduced how would this change the current service model?

Financial viability domain:

10. What new investment priorities are needed to provide thrombectomy for all eligible patients? *E.g. nursing staff, ambulance commissioning, angiography equipment*

11. What service developments outside of thrombectomy and stroke are there that might influence the availability of funding? *E.g. redesign of emergency care systems or changes in tariffs*

12. What would be care cost implications if patients are redirected to a thrombectomy centre? *E.g. hidden care costs for re-directed patients who do not receive a thrombectomy*

Sustainability domain and anything else:

13. What is needed for a sustainable model of thrombectomy provision across the NHS?

14. What other policy, strategic or financial developments are there that we have not covered/mentioned?
