# Supplementary material for: 10 years on from the landmark stroke thrombectomy trials, where are we now? A qualitative study examining professional views on the implementation of endovascular treatment for ischaemic stroke in England
Source: BMJ Open. 2025 Sep 17;15(9):e104126. doi: 10.1136/bmjopen-2025-104126 (PMC12458695; doi:10.1136/bmjopen-2025-104126)
Supplement: Supplementary file 2 [file bmjopen-2025-104126.reviewer_comments.pdf]

PEER REVIEW HISTORY

BMJ Open publishes all reviews undertaken for accepted manuscripts. Reviewers are asked to complete a checklist review form and are provided with free text boxes to elaborate on their assessment. These free text comments are reproduced below.

ARTICLE DETAILS

Title (Provisional)

Ten years on from the landmark stroke thrombectomy trials, where are we now? A qualitative study examining professional views on the implementation of endovascular treatment for ischaemic stroke in England

Authors

Simmonds, Rosemary L; Day, Jo; James, Martin; White, Phil; Price, Christopher; Shaw, Lisa; Ford, Gary; Pope, Catherine J

VERSION 1 - REVIEW

**Reviewer**	**1**
**Name**	**Rosewilliam, Sheeba**
**Affiliation**	**University of Birmingham, School of Health and Population Sciences**
**Date**	**10-Jul-2025**
**COI**	**None**

This study is well-designed, meticulously executed and reported clearly. It provides valuable insights into the complexity and challenges in the implementation of MT. A very informative read with excellent supportive quotes provided.

Below is some minor feedback to help improve the clarity and knowledge in some places for a reader:

1. Page 4 line 14: The statement could be made more specific with some statistics about how many hospitals do not provide or how many hospitals provide these services.

2. Page 4 line 32: Please clarify the 'selection processes' for a reader who is not an expert.

3. Page 6 line 23 and 24: The thematic networks approach and cross-cutting inductive themes have been mentioned but could be elaborated a bit more for a greater understanding (similar to how the Framework approach that has been clearly explained). For example, what does cross-cutting mean in this work and how was it done?

4. Page 7 line 16: Please introduce the cross-cutting themes earlier in the methods as above and explain it here, early in the results, where the themes are listed. This will improve

understanding of the cross-cutting of the themes ‘communications’ and ‘culture’ when the reader comes to read the complete description of this fourth theme later.

5. Page 8 line 37: The point about stroke being underplayed in ambulance training and dedicated (there is a comma here which makes it seem like there is a word missing) paid time for professional development sits within the ‘workforce’ theme. Perhaps the concept of ‘workforce training’ could be used as the link here so that the idea fits logically within this theme.

6. Page 9 line 15: It is mentioned that imaging and diagnostics were enhancing the speed of the MT pathway via an ASC referral but does not elaborate how this potentially happens. Perhaps a supportive quote that elaborates this idea and also breaks up the dense interpretative text would help readers to understand this point better.

7. Page 14 line 7: Professional protectionism is a complex concept that could be elaborated a bit more here, for example which aspects of professional protectionism are at play here.

8. Page 15 line 56 onwards till page 16 line 9: It would help to relook at this discussion para which is a summary of the points that were previously mentioned in the findings. Further knowledge or further recommendations based on the summarised findings in this paragraph would benefit the reader.

**Reviewer**	**2**
**Name**	**Jaroenngarmsamer, Tanaporn**
**Affiliation**	**Mahidol University**
**Date**	**12-Jul-2025**
**COI**	**None**

This is a qualitative study utilizing focus groups and interviews with 29 stakeholders involved in stroke care in England, aimed at identifying factors that influence the implementation of mechanical thrombectomy services for patients with ischemic stroke. The authors used both deductive and inductive methods to code the transcripts. They identified four themes related to the implementation of mechanical thrombectomy systems:

1. Workforce

2. Clinical care pathways

3. Services/systems

4. Cross-cutting themes: clinical communications and culture.

The first three themes are similar to the framework from the interview guide.

COMMENTS:

Methods

- Please clarify why data saturation was not included in the study design.

- If data saturation was not achieved, it may explain why certain aspects about thrombectomy systems, such as feedback data and research about new implementations, were not discussed.

Results

- The themes should be restated to better reflect key ideas/subthemes within the themes rather than simply replicating the framework from the interview guide. For example, use “clinical care pathways should be timely and seamless,” instead of “clinical care pathways.”

- Workforce: This theme can be divided into two subthemes covering personnel capacity and workforce education/training. The education component could incorporate the cultural aspects in the cross-cutting themes.

- Cross-cutting themes should be separated into two themes. Alternatively, the cultural element might be merged with the educational aspect under workforce theme, while the communication element (such as conference call) might be merged with the services/systems theme.

- On page 8, line 55, does “Eire” refer to the emergency system in Ireland? Please clarify.

- On page 9, line 7, the disorganized A&E should be emphasized according to the participant quote, before mentioning about bed capacity and repatriation.

- Figures 1 and 2 should be simplified, as they contain many overlapping ideas.

Discussion

- If SBAR was being proposed for application, consider discussing follow-up data or feedback regarding its implementation.

Reference

- The reference list, especially online references, should be reviewed and revised according to the journal’s citation style.

VERSION 1 - AUTHOR RESPONSE

Reviewer 1 Comments:
This study is well-designed, meticulously executed and reported clearly. It provides valuable insights into the complexity and challenges in the implementation of MT. A very informative read with excellent supportive quotes provided. Below is some minor feedback to help improve the clarity and knowledge in some places for a reader:
Response: We appreciate the very positive review and the helpful feedback to improve the paper for the reader.

#1. Page 4 line 14: The statement could be made more specific with some statistics

about how many hospitals do not provide or how many hospitals provide these services.
Response: We have provided more specific information of the number of hospitals that provide MT.

#2. Page 4 line 32: Please clarify the 'selection processes' for a reader who is not an expert.
Response: We have elaborated on what is meant by selection processes for someone not familiar with the pathway to thrombectomy treatment.

#3. Page 6 line 23 and 24: The thematic networks approach and cross-cutting inductive themes have been mentioned but could be elaborated a bit more for a greater understanding (similar to how the Framework approach that has been clearly explained). For example, what does cross-cutting mean in this work and how was it done?
Response: In the data analysis section we have further elaborated the thematic networks qualitative analysis tool, explained what cross-cutting means and how the cross cutting themes were generated and analysed in this study.

#4. Page 7 line 16: Please introduce the cross-cutting themes earlier in the methods as above and explain it here, early in the results, where the themes are listed. This will improve understanding of the cross-cutting of the themes 'communications' and 'culture' when the reader comes to read the complete description of this fourth theme later.
Response: We have identified that the latter two themes are cross-cutting and, in response to feedback from reviewer 2, we have re-labelled these in the paper as main themes to aid reader understanding.

#5. Page 8 line 37: The point about stroke being underplayed in ambulance training and dedicated (there is a comma here which makes it seem like there is a word missing) paid time for professional development sits within the 'workforce' theme. Perhaps the concept of 'workforce training' could be used as the link here so that the idea fits logically within this theme.
Response: We appreciate and agree with this feedback. In the paper we have added the concept of workforce training to improve the fit with this theme and edited the point made.

#6. Page 9 line 15: It is mentioned that imaging and diagnostics were enhancing the speed of the MT pathway via an ASC referral but does not elaborate how this potentially happens. Perhaps a supportive quote that elaborates this idea and also breaks up the dense interpretative text would help readers to understand this point better.
Response: Thank you for this suggestion and the following quote has been added. "So the magic wand would be, could we get the patients to have the right imaging in the acute stroke centres, [so] we can encourage more referrals into the comprehensive stroke centres..." (ID28)

#7. Page 14 line 7: Professional protectionism is a complex concept that could be elaborated a bit more here, for example which aspects of professional protectionism are at play here.
Response: We have elaborated further the aspects of the professional protectionism raised in this study by adding two quotes and further explanatory text as to how this is viewed as being at play by participants in the study.

#8. Page 15 line 56 onwards till page 16 line 9: It would help to relook at this discussion para which is a summary of the points that were previously mentioned in the findings. Further knowledge or further recommendations based on the summarised findings in this paragraph would benefit the reader.
Response: We have revised this paragraph on workforce to focus more on further recommendations based on the findings.

Reviewer 2 Comments:

#1. This is a qualitative study utilizing focus groups and interviews with 29 stakeholders involved in stroke care in England, aimed at identifying factors that influence the implementation of mechanical thrombectomy services for patients with ischemic stroke. The authors used both deductive and inductive methods to code the transcripts. They identified four themes related to the implementation of mechanical thrombectomy systems:
1. Workforce
2. Clinical care pathways
3. Services/systems
4. Cross-cutting themes: clinical communications and culture.
The first three themes are similar to the framework from the interview guide.
Response: Thank you for the summary of our study. We confirm the first three themes are deductive and the cross-cutting themes are inductive from our analysis. We have made this clearer in the revised paper.

#2. Please clarify why data saturation was not included in the study design and #3. If data saturation was not achieved, it may explain why certain aspects about thrombectomy systems, such as feedback data and research about new implementations, were not discussed.
Response: Thank you for noting this omission on how we designed the study. We have clarified in the methods section that we drew on Malterud et al., (2016) rather than data saturation for determining when we had achieved rich data collection within the resources and timetable available for this study. This could partially explain why feedback data was not highlighted as strongly by our participants. In addition, in England, Wales and Northern Ireland there is an established stroke care database (The Sentinel Stroke National Audit Programme - SSNAP) that provides timely information at all levels (local to national) and during our study there was, and continues to be, a strong national and regional focus in England on the continuous quality improvement of the implementation of stroke thrombectomy

drawing on information from the database.

#4. The themes should be restated to better reflect key ideas/subthemes within the themes rather than simply replicating the framework from the interview guide. For example, use “clinical care pathways should be timely and seamless,” instead of “clinical care pathways.”
Response: Thank you we considered this suggestion. At the time of writing we decided it was more appropriate to draw out the meanings within the narrative and quotes rather than imply them through lengthier theme headings/labels. We have decided to retain the short labels as three of the themes were deductively informed (therefore will align more with our topic guide as designed in our study), and as the points within each theme are not simplistic when we tried reworking the headings in line with this suggestion they became too verbose to capture all the meanings in the text.

#5. Workforce: This theme can be divided into two subthemes covering personnel capacity and workforce education/training. The education component could incorporate the cultural aspects in the cross-cutting themes.
Response: Given the limitations of the overall word count we decided that dividing this theme into two sub-themes would not be practical. We have edited the education component as advised by reviewer 1 #5 which addresses this point.

#6. Cross-cutting themes should be separated into two themes. Alternatively, the cultural element might be merged with the educational aspect under workforce theme, while the communication element (such as conference call) might be merged with the services/systems theme.
Response: Thank you for this suggestion and we have separated the two cross-cutting themes into two themes.

#7. On page 8, line 55, does “Eire” refer to the emergency system in Ireland? Please clarify.
Response: We have edited this to Dublin, Ireland to clarify the emergency system to which this relates.

#8. On page 9, line 7, the disorganized A&E should be emphasized according to the participant quote, before mentioning about bed capacity and repatriation.
Response: Thank you for suggesting and we have edited the text as follows:
“When hospital A&E departments experience extreme pressure of demand with ambulances queuing outside hospital A&E departments for general admissions, the inflow and outflow efficiency of the MT pathway is also impacted. Post MT procedure, rapid repatriation by ambulance to a local stroke centre is required to enable more stroke patients to be admitted and treated, but ambulances are less available under situations of heightened demand. The challenges of increased demand on ambulance services and hospital A&E departments, a lack of hospital bed capacity and staff shortages were seen to negatively impact on the capacity of the overall system to provide 24/7 stroke/MT treatment.”

#9. Figures 1 and 2 should be simplified, as they contain many overlapping ideas.
Response: We have edited the diagrams, in particular the text to give full phrase/names and directionality of the arrows which should help with readability. However, the thematic networks analytical method involves creating these types of network maps the goal of which is to show the visual representations of the complex relationships between different codes and themes (i.e. many overlapping ideas).

#10. If SBAR was being proposed for application, consider discussing follow-up data or feedback regarding its implementation.
Response: Thank you for this suggestion and we have elaborated this discussion point including two systematic review references to support the need for 'auditing' and confirming high fidelity implementation uptake in clinical settings.

#11. The reference list, especially online references, should be reviewed and revised according to the journal's citation style.
Response: The reference list has been reviewed and revised to be consistent with the citation style for the journal.
